# Monkeypox outbreak predominantly affecting men who have sex with men, Madrid, Spain, 26 April to 16 June 2022

**DOI:** 10.2807/1560-7917.ES.2022.27.27.2200471

**Published:** 2022-07-07

**Authors:** Jesús Iñigo Martínez, Elisa Gil Montalbán, Susana Jiménez Bueno, Fernando Martín Martínez, Alba Nieto Juliá, Jesús Sánchez Díaz, Natividad García Marín, Esther Córdoba Deorador, Antonio Nunziata Forte, Marcos Alonso García, Ana María Humanes Navarro, Laura Montero Morales, María José Domínguez Rodríguez, Manuel Carbajo Ariza, Luis Miguel Díaz García, Nelva Mata Pariente, Mercedes Rumayor Zarzuelo, Manuel José Velasco Rodríguez, Andrés Aragón Peña, Elena Rodríguez Baena, Ángel Miguel Benito, Ana Pérez Meixeira, María Ordobás Gavín, María Ángeles Lopaz Pérez, Araceli Arce Arnáez

**Affiliations:** 1 Directorate General of Public Health, Regional Ministry of Health of Madrid, Madrid, Spain

**Keywords:** Outbreak, monkeypox, men who have sex with men, public health, surveillance

## Abstract

Up to 22 June 2022, 508 confirmed cases of monkeypox (MPX) have been reported in the Madrid region of Spain, 99% are men (n = 503) with a median age of 35 years (range: 18–67). In this ongoing outbreak, 427 cases (84.1%) reported condomless sex or sex with multiple partners within the 21 days before onset of symptoms, who were predominantly men who have sex with men (MSM) (n = 397; 93%). Both the location of the rash, mainly in the anogenital and perineal area, as well as the presence of inguinal lymphadenopathy suggest that close physical contact during sexual activity played a key role in transmission. Several cases reported being at a sauna in the city of Madrid (n = 34) or a mass event held on the Spanish island of Gran Canaria (n = 27), activities which may represent a conducive environment for MPX virus spread, with many private parties also playing an important role. Because of the rapid implementation of MPX surveillance in Madrid, one of the largest outbreaks reported outside Africa was identified. To minimise transmission, we continue to actively work with LGBTIQ+ groups and associations, with the aim of raising awareness among people at risk and encouraging them to adopt preventive measures.

## Background

Monkeypox virus (MPXV) is an enveloped double-stranded DNA virus that belongs to the *Orthopoxvirus* genus of the *Poxviridae* family. There are two distinct genetic clades of the MPXV: the Central African (Congo Basin) clade and the West African clade. Monkeypox (MPX) is a viral zoonosis (a virus transmitted to humans from animals) with symptoms similar to those seen previously in smallpox patients, although it is clinically less severe. Transmission can occur from direct contact with the blood, bodily fluids, or cutaneous or mucosal lesions of infected animals. Human-to-human transmission can result from close contact with respiratory secretions, skin lesions of an infected person or recently contaminated objects [1]. Transmission via droplet respiratory particles usually requires prolonged face-to-face contact. With the eradication of smallpox in 1980 and subsequent cessation of smallpox vaccination, MPXV has emerged as the most important orthopoxvirus for public health [1].

Generally, outbreaks of MPX occur in countries from West and Central Africa [2,3] where, up to the 1980s, the transmission of the disease to humans has been mainly due to contact with animals. An increase in frequency was observed in the 1980s and, since the 1990s, there has been an increase in the number of secondary cases by contact with an infected person. Most cases observed outside of Africa were due to animal-to-human transmission, imported from an endemic country or associated with imported pets [4-6]. 

On 7 May 2022, the United Kingdom (UK) reported a case in a traveller from Nigeria. Between 13–16 May, six additional cases were reported, all of whom appeared to have been infected in London and self-identify as gay, bisexual or other men who have sex with men (MSM) [7,8]. On 18 May, Portugal reported the first 14 cases of MPX in men [9,10]. This is the first time that chains of person-to-person transmission of MPX have been reported in Europe, where no epidemiological links to West or Central Africa could be identified. Cases continue to be reported in several European countries [8,10,11].

### Outbreak detection

On 17 May 2022, a sexually transmitted diseases (STDs) clinic in Madrid reported seven suspected cases of MPX to the Regional Ministry of Health, which were microbiologically confirmed in vesicular lesions specimens. These seven cases represented the first occurrences of MPX in Spain and coincided in time with the two aforementioned outbreaks in the UK and Portugal. 

Here, we describe the clinical and epidemiological investigations of 508 cases reported from 17 May to 22 June, the first 5 weeks of the outbreak of MPX in Madrid region, and our containment strategy. 

## Methods

### Case detection

Cases were notified by STD clinics and public and private hospitals in the Madrid health network to the Surveillance System of the Regional Ministry of Health, between 17 May and 22 June 2022. 

The Regional Ministry of Health quantified the burden of MPX in the Madrid region (6,751,251 inhabitants [12]), described clinical and epidemiological characteristics of the disease and explored the mechanism of transmission in a non-endemic area. The outbreak investigation includes retrospectively identified cases with a symptom onset date before the initial outbreak notification. After detection of the first seven suspected cases of MPX, the Madrid Regional Ministry of Health established case definitions and developed a detailed and standardised questionnaire for the collection of clinical and epidemiological data based on available literature as well as guidelines for management of MPX disease in humans [4,13]. 

### Laboratory investigations

Vesicular lesion specimens were collected for each suspected case, and more specific samples were also taken depending on the patient's symptoms (urine, pharyngeal exudates, or other mucosal exudates). Specific real-time PCR for MPXV and genomic sequencing [14] were performed on the lesion samples.

The analysis of the samples was carried out in the National Reference Laboratory until May 27. After this date, laboratory capacity was increased and real-time PCR was implemented in five laboratories of the health network of Madrid; in one laboratory, genomic sequencing was also performed. A case-based epidemiological surveillance system was set up to allow analysis for the implementation of outbreak control measures.

### Case definitions

A suspected case was defined as a person with rash and presenting with at least one of the following symptoms: lymphadenopathy, fever, headache, asthenia, myalgia or arthralgia, since 15 March, once other pathologies had been ruled out. A probable case was someone meeting the case definition for a suspected case and one or more of the following in the 21 days before symptom onset: (i) travel history to a monkeypox endemic country, (ii) person (of any sexual orientation) who has had multiple or anonymous sexual partners, (iii) an epidemiological link to a probable or confirmed case. A confirmed case was someone with laboratory-confirmed MPX infection with a positive MPXV-specific PCR assay or sequencing result. The case classification used in this outbreak is similar to that established by the European Centre for Disease Prevention and Control (ECDC) [11] and the protocol subsequently established in Spain served to update the initial investigation form [15].

### Data collection

Data collection of the confirmed cases was performed by trained epidemiologists who completed a form that included sociodemographic characteristics, clinical manifestations, risk variables (sexual intercourse, attendance at mass events, saunas, and private sex parties), possible incubation period, contact history, personal medical history of interest (immunodeficiencies or other concurrent diseases and smallpox vaccination records) and contact with pets.

Close contacts of cases [15] were considered to include household members, sexual partners, social or work contacts, i.e. close (less than 2 meters in the same room) and prolonged contact (more than 3 hours [16]) with a confirmed case, and healthcare professionals who provided care without protective equipment from the symptom onset.

We defined a cluster as epidemiologically linked cases: identified cases who reported in the epidemiological survey that they had been in contact (i.e. household, sexual) with a confirmed case and provided their identity. Co-primary cases were those associated cases with a difference in onset of symptoms of less than 4 days.

## Results

### Timeline of the outbreak and descriptive epidemiology

Up to 22 June 2022, 802 suspected cases of MPX have been reported to the Surveillance System of the Community of Madrid, of which 595 cases (74.2%) were confirmed by real-time PCR, 183 cases (22.8%) were ruled out and 24 cases (3%) are still under investigation. This article analyses the data of 508 confirmed cases who were surveyed until 22 June, however, for the remaining 87 cases, the investigation is still ongoing.

The epidemic curve shows the evolution of the outbreak, with the first cases appearing at the end of April (26 and 27 April), and 60 additional cases presenting clinical symptoms before the outbreak declaration in Madrid on 17 May (Figure).

**Figure:**
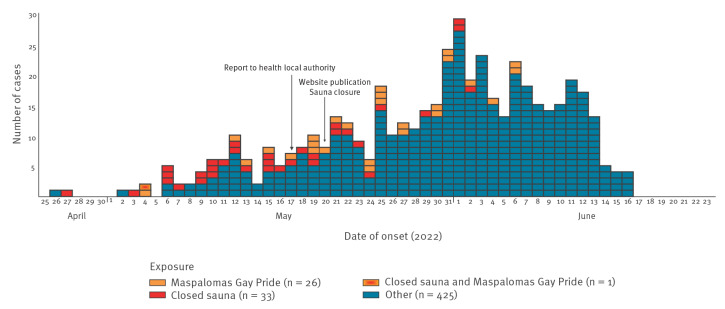
Epidemic curve of confirmed cases of monkeypox by onset of symptoms and likely location of infection, Madrid, Spain, 26 April–16 June 2022 (n = 485)^a^

^a^ Between 17 May and 22 June, 508 cases were reported, with symptom onset dates ranging from 26 April to 16 June. Data on symptom onset were missing for 23 cases.

Black arrows denote dates when a clinic of sexually transmitted diseases (STDs) in Madrid reported to the Regional Ministry of Health the first 7 suspected cases of monkeypox (17 May 2022) and when an official website containing information on epidemiology and control measures was launched (20 May 2022). Other sources of exposure (blue bar) include: dating through social networks, sexual encounters in private flats, cruising areas and bars, parties, large events and saunas different from the closed one (red bar).

Confirmed cases were mostly male (99%), with a median age of 35 years (interquartile range (IQR): 12; range: 18–67) (Table). 

**Table:** Characteristics of confirmed cases of monkeypox, Madrid, Spain, 26 April–16 June 2022 (n = 508)

Characteristics	Confirmed cases (n = 508)			
	n			%
Sex				
Men		503	99.0	
Women		5	1.0	
Age (years)				
< 20		4	0.8	
20–29		115	22.6	
30–39		211	41.5	
40–49		129	25.4	
50–59		43	8.5	
60–69		6	1.2	
Symptoms^a^				
Exanthema		498	98.0	
Fever		324	63.8	
Lymphadenopathy		311	61.2	
Asthenia		238	46.9	
Myalgia		185	36.4	
Headache		162	31.9	
Odynophagia		143	28.1	
Proctitis		81	15.9	
Rash location (n = 498)				
Anogenital and/or perineal area		359	72.1	
Legs and/or arms		222	44.6	
Face		177	35.5	
Chest and/or abdomen		159	31.9	
Back		132	26.5	
Palms and/or plants		124	24.9	
Lymphadenopathy location (n = 311)				
Inguinal		225	72.3	
Cervical		75	24.1	
Submandibular		42	13.5	
Axillary		7	2.3	
Retroauricular		7	2.3	
Medical history				
HIV infection		225	44.3	
PrEP		56	11.0	

PrEP: pre-exposure prophylaxis.

^a^ Several symptoms could be reported by each case.

The predominant clinical features were the presence of rash (n = 498; 98.0%), located in 72.1% (n = 359) of the cases in the anogenital and/or perineal area, in different stages of evolution, together with lymphadenopathy (n = 311; 61.2%), with the inguinal location being the most frequent (n = 225; 72.3%). Fever was present in 63.8% (n = 324) of cases. Other frequent symptoms were asthenia (n = 238; 46.9%) and myalgia (n = 185; 36.4%) (Table). For most cases, clinical symptoms were mild, although 19 cases (3.7%) required hospitalisation. Of these, four cases had dates of admission before the diagnosis of the first case in Madrid, indicating that the reasons for admission were not related to MPX complications (two cases of febrile syndrome with rash and two cases of proctitis under study). Seven of the 19 were hospitalised for MPX complications (parapharyngeal abscess, mouth ulcers, bacterial superinfection) and for the other eight cases, data were not available. 

Of 508 cases reporting medical history, 44.3% (n = 225) had HIV infection and 11% (n = 56) were on PrEP treatment.

A total of 427 cases (84.1%) reported condomless sex or sex with multiple partners, within the 21 days before the onset of symptoms, who were predominantly MSM (n = 397; 93%). Forty-one cases (8.1%) declared not having sex without a condom, and 40 (7.9%) did not answer. Four hundred and eight cases (80.3%) were unaware of or reported no contact with a known case of MPX.

### Contacts and exposure location

Forty-five clusters with 96 linked cases were identified, ranging from two to four cases per cluster. Twenty-six clusters have 54 (51%) co-primary cases (epidemiologically linked cases with a difference in onset of symptoms of less than 4 days). Nineteen transmission chains have been identified, with 42 cases: 20 are primary cases, 21 secondary cases (all close physical contact during sexual activities, 13 between household members and eight with non-household members) and 1 tertiary case. Thirty-eight cases have a history of international travel (including Italy (n = 8), Portugal (n = 7), Belgium (n = 4), Germany (n = 4), the UK (n = 3) and Peru (n = 2)); travel to Austria, Greece, Hungary, Czech Republic, Turkey, Colombia, Cuba, Ecuador, Venezuela, or United States was reported by one case each. No cases reported travel to Africa.

We investigated the possible source of exposure for the 508 confirmed cases. Dating through social networks (dating apps) was reported by 289 (56.9%) cases, which led to sexual encounters in private flats, cruising areas and bars; the majority (n = 206; 71.3%) had these encounters in private flats. Attendance at mass events and parties was reported by 73 (14.4%) cases, particularly the Gay Pride Maspalomas festival in Gran Canaria, Spain, from 5–15 May 2022 (n = 27). Condomless sex or sex with unknown partners in saunas was reported by 56 (11%) cases, of whom 34 attended the same establishment.

A total of 1,100 contacts have been reported in the epidemiological investigation, of whom 536 (48.7%) were unknown sex partners, 346 (31.5%) were family or household members, 176 (16%) were known sex partners, 26 (2.4%) were health personnel and 16 (1.4%) were social or work contacts. A total of 153 individuals have been included as contacts under surveillance (all other known contacts declined to participate in follow-up), and secondary transmission has been detected in the household setting in 13 close household contacts; two were women.

Information on pets was collected from 390 cases, of whom 72 (18.5%) live with pets, mostly dogs (n = 45; 62.5%) and cats (n = 29; 40.3%).

### Outbreak control measures

After the first suspected cases of MPX in Madrid were reported on 17 May 2022 to the Madrid Regional Ministry of Health, the initial case definition and sample referral network were established, and recommendations on prevention and management of suspected cases and identification of contacts were provided.

For all cases, including those suspected, recommendations were made for home isolation under clinical and epidemiological surveillance, as well as the use of surgical masks when sharing the same room to avoid potential droplet transmission. Avoiding contact with domestic animals was also recommended. These measures were to be maintained until the skin lesions were crusted and all scabs had fallen off. Close contacts were followed up for 21 days by trained epidemiologists once per week; they were informed about the symptoms associated with the disease and the need to take strict precautions, including advice to abstain from sexual intercourse and close physical contact during this period.

The Regional Ministry of Health published documents with recommendations on occupational risk prevention for healthcare professionals [17] and a procedure for channelling the referral of samples to laboratories with the capacity to respond in less than 24 hours.

A dedicated official website containing information on epidemiology and control measures was launched on 20 May 2022 [18]. Information materials with infographics for cases and contacts and frequently asked questions and answers were set up, which included a specific email address, for the submission of queries from citizens and professionals. On the same date, 16 associations working with LGBTIQ+ groups were informed of the outbreak and provided with recommendations for early diagnosis. In the following week, a face-to-face meeting was held between public health authorities and these associations with the aim of extending and strengthening prevention and control measures. Collaboration with owners of premises where sex with multiple partners can take place, with the aim of reaching agreement on codes of good hygiene practice. Of note, a sauna in the centre of Madrid where 34 of the confirmed male cases had engaged in sexual contact with other men was identified, and on 20 May 2022, the public health authorities closed it down temporarily.

## Discussion

This epidemiological study reports on 508 confirmed human cases of MPX during the first 5 weeks of the Madrid outbreak in 2022. In this large outbreak, the occurrence of almost all cases (99%) in MSM, together with the predominant location of the rash in the genital, perineal or perianal area and the presence of lymphadenopathy in the inguinal region, indicate that close physical contact during sexual activities has been highly involved in the transmission of the infection in this outbreak.

At the beginning of the outbreak, a high percentage of cases attended at the same sauna in the city of Madrid (34 of the 56 cases reporting sauna use), and the public health authorities proceeded to close down the sauna as a precautionary measure. This type of sauna can be a place of exposure to condomless sex with multiple unknown partners. In addition, 73 cases attended large events and parties, including the Maspalomas Gay Pride festival in Gran Canaria in May, which welcomed between 25,000 and 30,000 visitors from outside the island [19].

The travel history to Maspalomas, which has been observed both in the cases diagnosed in Madrid and in cases diagnosed in the Canary Islands, as well as in other European countries [20], together with the high number of sexual partners reported by the cases during their stay, suggests the possibility of a single outbreak of European predominance. 

Among the confirmed cases, 21 secondary cases and one tertiary case have been identified. Their exposure to MPXV preceded the diagnosis and isolation of their index cases. However, once the isolation of the cases was in place, no secondary cases appeared among the known contacts of the first 200 cases who have already completed the 21-day follow-up period. These findings differ from the higher secondary attack rates found in household contacts in other MPX outbreaks studied, particularly in Africa [6].

Sequencing of the viruses isolated from two cases in Madrid shows that they belong to the West African clade [14], which appears to cause less severe disease compared with the Central African clade [11,13]. Clinical manifestations were mild in almost all cases. Nineteen cases, 3.7% of the total number of confirmed cases, were hospitalised, seven on account of complications associated with MPX.

HIV infection and its level of control may modify the severity and duration of the clinical signs [11,21]. Although 44.3% of cases reported being HIV-positive, there has been no observed increase in severity. This is likely because of the adequate immune control with undetectable viral load in almost all cases (data not shown). A MPX outbreak studied in Nigeria in 2017–18 [5] resulted in seven deaths among 118 confirmed cases (6%), of whom four were HIV-positive with poor disease control. The high percentage of HIV-positive cases in our report could be because of the ease of patient access to the Spanish healthcare system, and therefore a high testing rate. However, protocols have been established by the Regional Ministry of Health in Madrid that any suspected case should be referred to a hospital emergency department to facilitate clinical and microbiological diagnosis.

There is some debate about the protection provided by the smallpox vaccine against disease occurrence and severity [3,4]. In Spain, smallpox vaccination was discontinued in the early 1980s, so people under 40 years of age would not have been vaccinated. In this outbreak, the median age of cases was 35 years, and 65% were aged under 40 years, so they would be less protected than other age groups against MPX, but vaccination status was unknown for most cases. Thus far, however, this has not translated into a higher severity of cases.

Our study has some limitations. One limitation pertains to data collection. Although in most medical records we find that HIV-positive cases have an undetectable viral load, these data have not been collected in the survey. In addition, the suspected MPX cases who were ruled out were not included in the study, so we did not have information on their final diagnosis. Also, a high number of cases did not know or could not prove whether they had been vaccinated against smallpox. Another challenge of the study has been to accurately determine the incubation period of the disease in many of the cases, as most are mild, paucisymptomatic cases with small and few skin lesions; this has contributed to the lack of awareness of the disease on the part of the cases and of the inadvertent exposure to MPX on the part of their contacts. In addition, sexual intercourse with multiple anonymous partners makes it difficult to determine a single exposure. 

## Conclusion

Because of the rapid implementation of MPX surveillance in Madrid, one of the largest outbreaks reported outside Africa thus far was identified. Considering the transmission pattern of this outbreak and the fact that most of the contacts are unknown, which is an added difficulty for the knowledge and control of the outbreak, it is expected that the number of cases may continue to increase in the coming weeks. To minimise further transmission, we continue to actively work with LGBTIQ+ groups and associations, with the aim of raising awareness among people at risk and encouraging them to adopt preventive measures. In addition, it is important to raise awareness among physicians as this outbreak may not be limited to the LGBTIQ+ community. 
